# Tmem88 plays an essential role in pharyngeal pouch progenitor specification by inhibiting Wnt/β-catenin signaling

**DOI:** 10.1093/lifemedi/lnad044

**Published:** 2023-11-17

**Authors:** Jingwen Liu, Liping Yang, Zidong Lu, Qiang Wang

**Affiliations:** State Key Laboratory of Membrane Biology, Institute of Zoology, University of Chinese Academy of Sciences, Chinese Academy of Sciences, Beijing 100101, China; Innovation Centre of Ministry of Education for Development and Diseases, The Sixth Affiliated Hospital, School of Medicine, South China University of Technology, Guangzhou 510006, China; Innovation Centre of Ministry of Education for Development and Diseases, The Sixth Affiliated Hospital, School of Medicine, South China University of Technology, Guangzhou 510006, China; Innovation Centre of Ministry of Education for Development and Diseases, The Sixth Affiliated Hospital, School of Medicine, South China University of Technology, Guangzhou 510006, China

**Keywords:** Tmem88, Wnt/β-catenin, pharyngeal pouch, progenitor, specification

## Abstract

Pharyngeal pouches, which are endodermal outpockets that segment the pharyngeal arches, play a crucial role in the development of craniofacial skeletons in vertebrate embryos. Our previous study successfully identified pharyngeal pouch progenitors (PPPs) in zebrafish embryos and emphasized the significance of BMP2b signaling in their specification. However, the specific mechanism by which these progenitors originate from endodermal cells remains largely unknown. Here we found that the pharmacological activation of Wnt signaling pathway disrupts the emergence of PPPs and subsequently hinders the formation of pharyngeal pouches. Moreover, we have identified the expression of *tmem88a* and *tmem88b* (collectively known as *tmem88a/b*) in PPPs during the early-somite stages. Furthermore, the deficiency of *tmem88a/b* leads to an excessive accumulation of β-catenin in both the cytoplasm and nucleus of endodermal cells that are intended to differentiate into PPPs. Importantly, suppressing the hyperactivation of Wnt/β-catenin signaling through pharmacological treatment, the defects in PPP specification in *tmem88a/b*^−/−^ mutants are successfully rescued. In summary, our findings establish a clear connection between the specification of PPPs and the regulation of Wnt signaling mediated by Tmem88. These results underscore the pivotal role of Tmem88 in the development of pharyngeal pouches.

## Introduction

Pharyngeal pouches are endodermal outpockets that occur between the pharyngeal arches in vertebrate embryos [1]. These pouches play a crucial role in the development of the pharyngeal skeleton, the specification of arch-associated ganglia, and the morphogenesis of the arch arteries by expressing various signaling molecules, including FGFs, BMPs, and PDGFs [2–6]. Furthermore, the pouches contribute to the formation of several endodermal organs in the head and neck region, such as the middle ear cavity, Eustachian tube, palatine tonsil, thymus, and parathyroid [7]. Notably, disorders like DiGeorge syndrome and Branchio-Oto-Renal syndrome, which are characterized by underdeveloped parathyroid glands and thymus, result from abnormal development of the third and fourth pouches in humans, highlighting the significance of pharyngeal pouch development [8]. In our previous study, we demonstrated the emergence of *nkx2.3*^+^ pharyngeal pouch progenitors (PPPs) in the outermost part of the pharyngeal endoderm during early-somite stages. The development of these progenitors relies on BMP2b signaling from the adjacent ectoderm [9]. However, the molecular mechanisms underlying the specification of PPPs remain largely unknown.

Transmembrane protein 88 (TMEM88) was initially identified in 2010 as a disheveled-binding protein [10]. Due to its C-terminal PDZ-binding motif, TMEM88 is hypothesized to inhibit Wnt signaling by interacting with Dvl proteins and/or sequestering the Wnt signalosome to multivesicular bodies, which subsequently fuse with the lysosome [10, 11]. TMEM88 serves as an inhibitory factor of Wnt/β-catenin signaling and is crucial for cardiomyocyte differentiation [12] and the proper balance between cell cycle progression and cell fate determination during pharyngeal arch artery development [13]. Previous reports have indicated the expression of *tmem88a* and *tmem88b* (collectively referred to as *tmem88a/b*) in the lateral plate mesoderm of early-somite stage zebrafish embryos, which give rise to the heart and blood vessels [12]. PPPs are located adjacent to the lateral plate mesoderm and share a developmental stage with pericardial precursors [9]. However, the expression of *tmem88a/b* in PPPs and their role in PPP specification by suppressing the Wnt/β-catenin signaling pathway have not been thoroughly explored.

In this study, we conducted pharmacological experiments and observed that overactivation of Wnt/β-catenin signaling during the early-somite stage significantly reduced the number of PPPs and led to severe defects in pharyngeal pouch formation. Subsequently, fluorescence *in situ* hybridization experiments confirmed the expression of both *tmem88a* and *tmem88b* in the *nkx2.3*^+^ PPPs. Importantly, loss-of-function analyses revealed the essential role of *tmem88a/b* in the emergence of PPPs within the pharyngeal endoderm by restricting Wnt/β-catenin signal activation. Thus, our findings uncover the involvement of Tmem88-mediated Wnt signal restriction in ensuring appropriate control of cell fate during pharyngeal pouch development.

## Results

### Hyperactivation of Wnt signaling disrupts pharyngeal pouch formation

**Figure 1.**
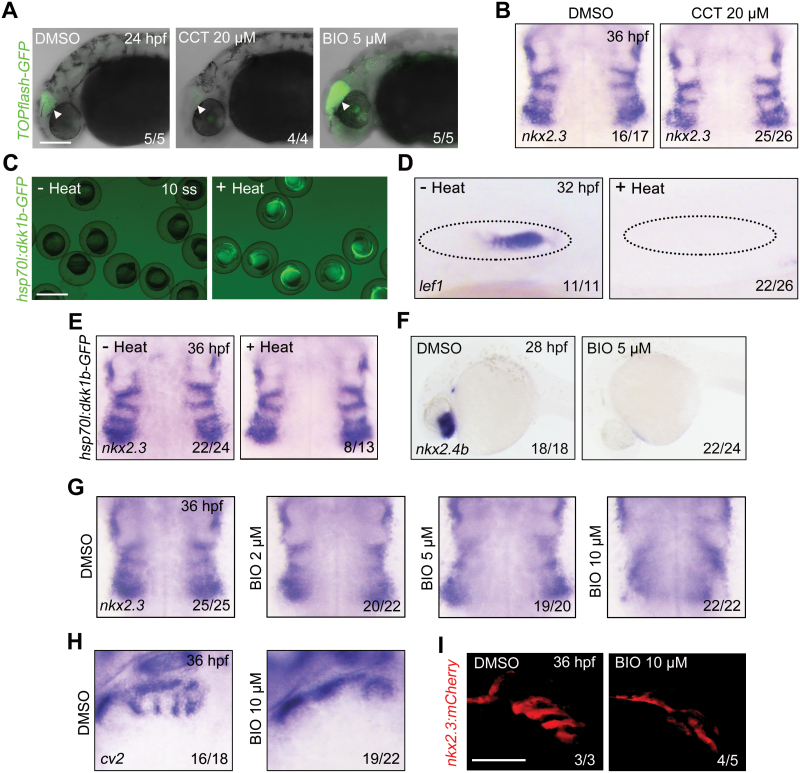
**BIO treatment disturbs the morphogenesis of pharyngeal pouches.** (A) *Tg(TOPflash-GFP)* embryos were treated with CCT036477 or BIO from bud stage to 24 hpf. Scale bar, 100 μm. CCT, CCT036477. (B) *In situ* hybridization analysis of *nkx2.3* expression in embryos treated with DMSO or CCT036477 from the bud stage to 17 ss. (C) Heat shock-induced expression of Dkk1b in *Tg(hsp70l:dkk1b-GFP)* embryos. Heat shock was performed at the bud stage, and GFP fluorescence was imaged at 10 ss. Scale bar, 200 μm. (D, E) *Tg*(*hsp70l:dkk1b-GFP*) embryos were heat-shocked at the bud stage and harvested for *in situ* hybridization with the *lef1* (D) or *nkx2.3* (E) probe. (F) Comparative *in situ* hybridization analysis of *nkx2.4b* expression in wild-type embryos treated with or without BIO from the bud stage. (G, H) Wild-type embryos were exposed to different concentrations of BIO from the bud stage to 17 ss and harvested at 36 hpf for *in situ* hybridization with the *nkx2.3* (G) or *cv2* (H) probe. The ratios of affected embryos were indicated. (I) *Tg*(*nkx2.3:mCherry*) embryos were treated with DMSO or 10 μM BIO from the bud stage to 17 ss. Subsequently, these embryos were harvested at 36 hpf for *in vivo* confocal imaging. Scale bars, 100 μm.

In our previous study, we conducted a screening using chemical inhibitors to identify the essential signaling pathways involved in the emergence of PPPs [9]. Interestingly, although *Tg(TOPflash-GFP)* transgenic embryos treated with 20 μM CCT036477, a selective suppressor of Wnt signaling, exhibited significantly reduced expression of the β-catenin/TCF-mediated reporter [14], embryos that had undergone similar treatments showed normal pouch formation (Fig. 1A and 1B) [9]. This suggests that Wnt signaling is not necessary for PPP specification. To further validate this observation, we induced the overexpression of Dickkopf-related protein 1b (Dkk1b), a secreted Wnt inhibitory factor, in *Tg(hsp70l:dkk1b-GFP)* embryos through heat shock (Fig. 1C) [15]. Dkk1b overexpression led to significantly decreased expression of *lef1* in the developing neuromasts (Fig. 1D), corroborating previous report [16]. However, after heat shock treatment, *Tg(hsp70l:dkk1b-GFP)* embryos exhibited no obvious defects in the expression of the pharyngeal pouch marker *nkx2.3* at 36 h postfertilization (hpf) (Fig. 1E).

On the other hand, it is intriguing to explore whether hyperactivation of the Wnt signaling pathway could interfere with pouch formation. To investigate this, we treated wild-type embryos with BIO, a selective chemical activator of Wnt/β-catenin signaling, from the bud stage until the 17-somite stage (17 ss) (Fig.1A) [17]. Consistent with previous reports [14, 16, 18], the expression of *nkx2.4b* was dramatically decreased upon BIO treatment (Fig. 1F). *In situ* hybridization experiments clearly demonstrated a dose-dependent reduction in the expression of *nkx2.3* in the pharyngeal region of BIO-treated embryos (Fig. 1G). Similarly, the expression of another marker for pouch endoderm, *crossveinless 2* (*cv2*), was significantly decreased upon BIO treatment (Fig. 1H). To evaluate the morphological changes in the pharyngeal pouches, we exposed *Tg*(*nkx2.3:mCherry*) transgenic embryos to BIO and observed a dramatic disruption in the formation of endodermal pouches in embryos with overactivated Wnt signaling (Fig. 1I). Taken together, these findings indicate that excessive activation of Wnt signaling during the early-somite stages leads to the aberrant development of pouch tissues.

### Hyperactivation of Wnt signaling attenuates PPP specification

**Figure 2.**
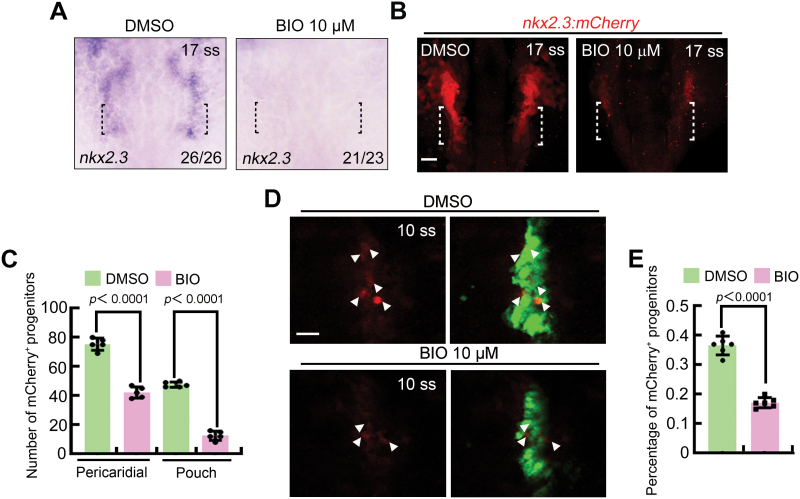
**Excessive Wnt signal compromises PPP specification.** (A) Expression of *nkx2.3* in embryos treated with DMSO or BIO. The black dotted lines indicate the region where the PPPs are located. (B, C) Representative confocal sections showing *Tg*(*nkx2.3:mCherry*) embryos treated with DMSO or BIO (B). The PPPs are indicated by white dotted lines. Scale bar, 20 μm. Quantitative analysis of PPP cell numbers is shown in (C). The group values are expressed as mean ± SD. (D, E) Confocal images illustrating the specification of *nkx2.3*^+^ pouch progenitors from the pharyngeal endoderm. White arrowheads indicate the presence of GFP^+^/mCherry^+^ cells in *Tg*(*nkx2.3:mCherry;sox17:GFP*) embryos (D). Scale bar, 20 μm. Ratio of mCherry^+^ PPPs to the GFP^+^ pharyngeal endoderm of embryos treated with DMSO or BIO was achieved from three independent assays (E). The group values are expressed as mean ± SD.

The findings mentioned above highlight the significant impact of aberrant Wnt signaling activation during somitogenesis on pouch formation in later stages of development. Therefore, we next sought to investigate the potential influence of Wnt signal overactivation on the emergence of PPPs. At the 17 ss, the *nkx2.3*-expressing bilateral clusters consist of two distinct cell types: pericardial precursor cells in the anterior region and PPP cells in the posterior region (Fig. 2A) [9]. Interestingly, we observed a notable reduction in the expression of *nkx2.3* in both the anterior and posterior regions of BIO-treated embryos (Fig. 2A), indicating compromised formation of pericardial precursors and PPPs. Consistently, BIO-treated *Tg*(*nkx2.3:mCherry*) transgenic embryos exhibited a significant decrease in the number of pericardial precursors, particularly PPPs (Fig. 2B and 2C). Furthermore, we examined the specification of *nkx2.3*^+^ pouch progenitors derived from the pharyngeal endoderm in *Tg*(*nkx2.3:mCherry;sox17:GFP*) embryos at the 10 ss. We observed a reduction in the ratio of mCherry^+^ cells to the GFP^+^ pharyngeal endoderm of BIO-treated embryos when compared to that of the control group (Fig. 2D and 2E), implying impaired pouch progenitor formation. These findings strongly suggest that excessive activation of Wnt signaling impedes the specialization of PPPs, ultimately resulting in a severe loss of pouch endoderm.

### Tmem88 is required for PPP specification

**Figure 3.**
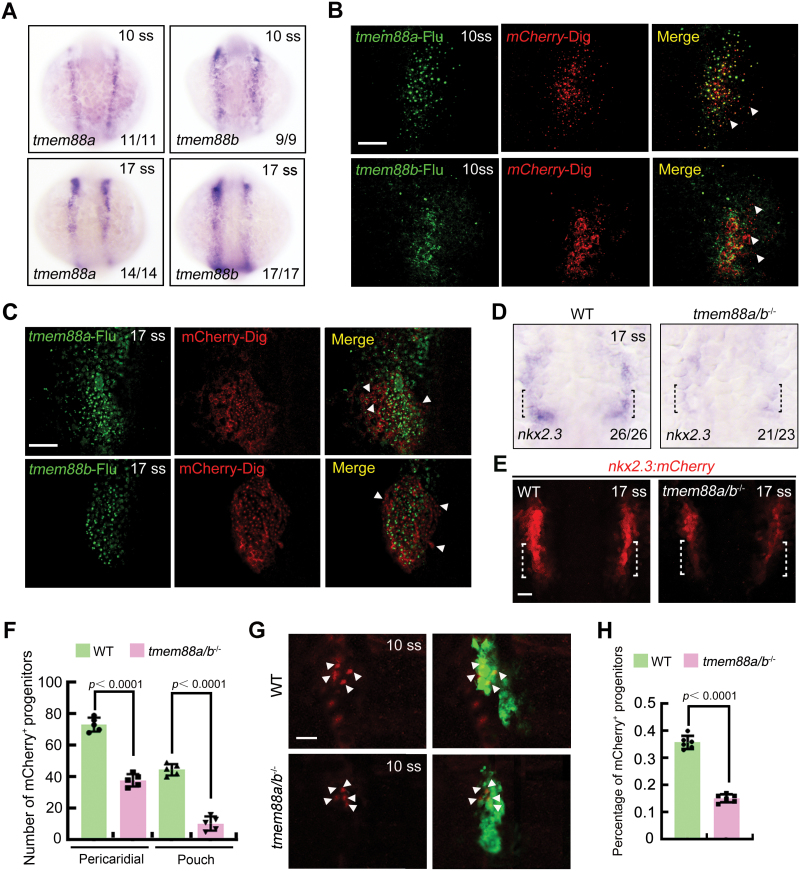
**Tmem88 deletion severely impedes PPP specification.** (A) *In situ* hybridization of *tmem88a* and *tmem88b* in wild-type embryos. (B, C) Double-color fluorescence *in situ* hybridization showing the expression of *mCherry* (red) and *tmem88a* or *tmem88b* (green) in *Tg*(*nkx2.3:mCherry*) embryos at the 10 (B) and 17 ss (C). The white arrow heads indicate the mCherry^+^ PPPs that do not express *tmem88a* or *tmem88b*. Scale bar, 50 μm. (D) Expression of *nkx2.3* in wild-type and *tmem88a/b*^−/−^ embryos at the 17 ss. The black dotted lines indicate the region where the pouch progenitors are located. (E, F) Representative confocal sections showing mCherry^+^ PPPs in wild-type and *tmem88a/b*^−/−^ embryos at 17 ss (E). The PPPs are indicated by white dotted lines. Scale bar, 50 μm. Quantification of the number of pericardial and pouch progenitors positive for mCherry in wild-type and *tmem88a/b*^−/−^ mutants is shown in (F). The group values are expressed as mean ± SD. (G, H) Requirement of Tmem88 for pouch progenitor specification. Wild-type and *tmem88a/b*^−/−^ mutant embryos in the *Tg*(*nkx2.3:mCherry;sox17:GFP*) background were imaged at 10 ss (G). Scale bar, 20 μm. Ratio of mCherry^+^ PPPs to the GFP^+^ pharyngeal endoderm of wild-type or *tmem88a/b*^−/−^ embryos was quantified from three independent assays (H). The group values are expressed as mean ± SD.

Given the role of Tmem88 in inhibiting Wnt signaling in zebrafish embryos [12, 13], we first examined the expression of *tmem88a* and *tmem88b* during the 10 and 17 ss using *in situ* hybridization. We observed robust detection of *tmem88a* and *tmem88b* transcripts in bilateral clusters within the pharynx at these early-somite stages (Fig. 3A). To determine the expression of *tmem88a/b* in PPPs, we conducted double fluorescence *in situ* hybridization using mCherry and *tmem88a* or *tmem88b* probes in *Tg*(*nkx2.3:mCherry*) embryos. The results depicted in Fig. 3B and 3C clearly demonstrate the expression of both *tmem88a* and *tmem88b* transcripts in cells located in both the anterior and posterior regions of the mCherry-expressing bilateral clusters during somitogenesis, indicating that *tmem88a/b* are expressed in pericardial precursors and PPPs. Interestingly, we noted that some of these mCherry^+^ precursors do not express *tmem88a/b*, potentially suggesting differences in their developmental stages or cellular heterogeneity.

As *tmem88a* and *tmem88b* function redundantly in zebrafish embryos [13], we then investigated whether Tmem88 regulates the specification of PPPs in *tmem88a/b*^−/−^ mutants. We observed a significant reduction in the expression of *nkx2.3* in *tmem88a/b*^−/−^ mutants at the 17 ss (Fig. 3D). Furthermore, *Tg*(*nkx2.3:mCherry*) embryos lacking *tmem88a/b* exhibited a decreased number of pericardial progenitors and PPPs (Fig. 3E and 3F). Consistent with these observations, the ration of mCherry^+^ cells to the GFP^+^ pharyngeal endoderm was significantly reduced in *tmem88a/b*^−/−^ mutants in the *Tg*(*nkx2.3:mCherry;sox17:GFP*) background, indicating impaired specification of PPPs from the pharyngeal endoderm (Fig. 3G and 3H). These findings provide compelling evidence that Tmem88 is essential for the specification of PPPs.

### The pharyngeal pouches can develop independently of tmem88a/b

**Figure 4.**
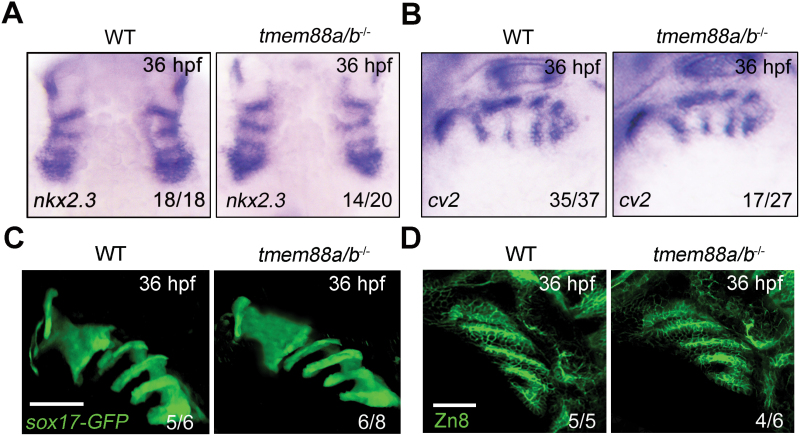
**Tmem88 deletion has minor effect on pharyngeal pouch morphology.** (A, B) The expression of *nkx2.3* (A) and *cv2* (B) in wild-type and *tmem88a/b*^−/−^ mutants at 36 hpf. (C) Wild-type and *tmem88a/b*^−/−^ mutant embryos in the *Tg*(*sox17:GFP*) background were imaged at 36 hpf. Scale bars, 20 μm. (D) Zn8-labeled pharyngeal pouches in both wild-type and *tmem88a/b*^*−/−*^ mutants at 36 hpf. Scale bars, 50 μm.

To further investigate the impact of *tmem88a/b* deficiency on pharyngeal pouch formation during later developmental stages, we conducted an examination of pouch endoderm markers in *tmem88a/b*^−/−^ mutants at 36 hpf. Surprisingly, despite the clear requirement for *tmem88a/b* in PPP emergence, the expression of *nkx2.3* and *cv2* in the pharyngeal region was minimally disrupted in *tmem88a/b*^−/−^ embryos (Fig. 4A and 4B). Additionally, the development of pouch structures proceeded normally in the absence of *tmem88a/b* (Fig. 4C and 4D). These observations suggest that even without *tmem88a/b*, although there is a significant reduction in PPPs, the pouch endoderm is still capable of forming. This could be attributed to accelerated proliferation of the remaining precursor cells or the presence of an alternative cell population that compensates for the absence of PPPs during later developmental stages.

### Tmem88 regulates PPP specification by suppressing Wnt/β-catenin signaling

**Figure 5.**
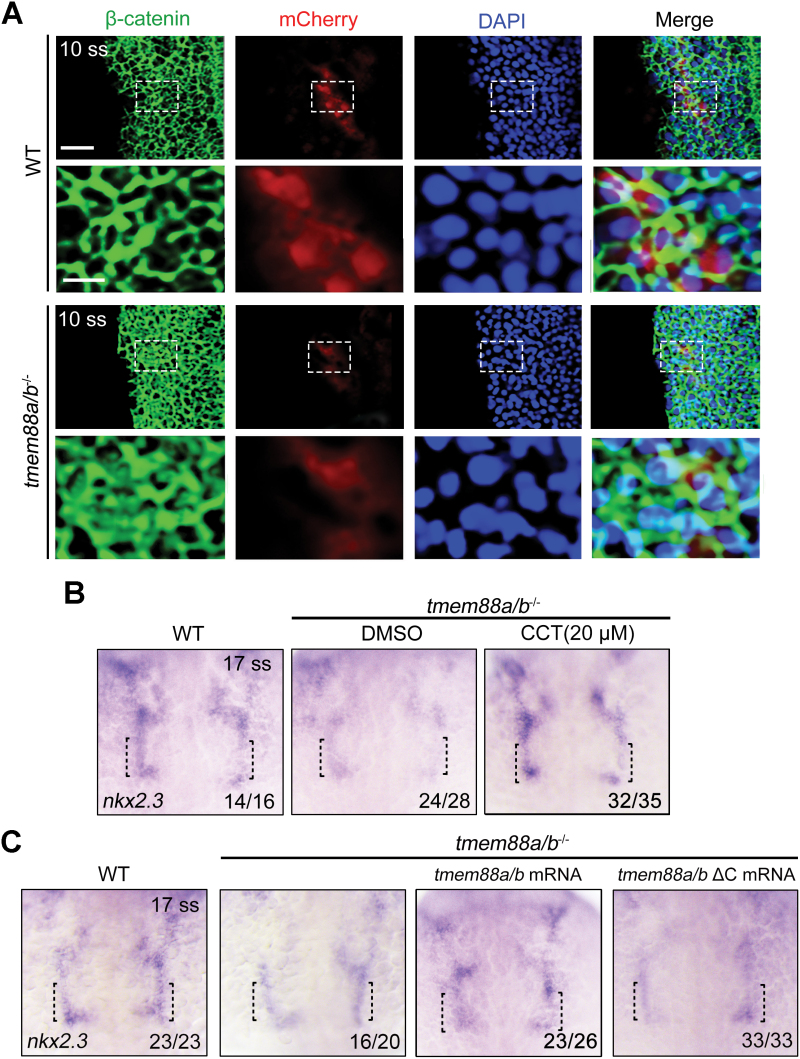
**Tmem88 promotes PPP specification through inhibiting Wnt/β-catenin signaling.** (A) The subcellular localization of β-catenin in PPP cells of both wild-type and *tmem88a/b*^−/−^ embryos in the *Tg*(*nkx2.3:mCherry*) background. Embryos were stained with the indicated antibodies. Nuclei were counterstained with DAPI (blue). The boxed area in the upper image (scale bar, 20 μm) is presented at a higher magnification in the corresponding lower image (scale bar, 100 μm). (B) Expression of *nkx2.3* in wild-type or *tmem88a/b*^−/−^ embryos treated with or without 20 μM CCT036477. (C) Expression of *nkx2.3* in wild-type or mutant embryos injected with indicated mRNAs at 17 ss.

The above analyses have provided compelling evidence that excessive activation of the Wnt signaling pathway leads to impaired specification of PPPs. Given the mitigating effect of *tmem88a/b* deletion on PPP defects, our next aim was to investigate the potential role of Tmem88 in PPP emergence by modulating Wnt/β-catenin signaling. We initially examined the subcellular localization of β-catenin in PPP cells of both wild-type and *tmem88a/b*^−/−^ embryos at 10 ss. Immunofluorescence staining revealed an increased accumulation of β-catenin in the cytoplasm and nucleus of *tmem88a/b*-depleted mCherry^+^ PPPs compared to control cells (Fig. 5A), indicating aberrant activation of the Wnt/β-catenin pathway. Based on this finding, we hypothesized that suppressing the overactivated Wnt/β-catenin signaling in *tmem88a/b*^−/−^ mutants could rescue the defects in PPP specification. To test this hypothesis, *tmem88a/b*^−/−^ embryos were treated with 20 μM of the Wnt signaling inhibitor CCT036477 from the bud stage, and we observed that this pharmacological treatment eliminated the reduction in *nkx2.3* expression caused by *tmem88a/b* depletion (Fig. 5B). In a previous study, we demonstrated the essential role of the C-terminal PDZ-binding motif in Tmem88a or Tmem88b for inhibiting Wnt signaling [14]. Consistently, when wild-type *tmem88a/b* mRNAs were injected into the mutants, *nkx2.3* expression was significantly restored (Fig. 5C). Conversely, overexpression of C-terminal truncated Tmem88a/b proteins lacking the PDZ-binding motifs in mutant embryos did not have a noticeable rescue effect on *nkx2.3* expression (Fig. 5C). Taken together, these results unequivocally demonstrate that Tmem88 primarily promotes PPP specification by regulating the activity of the Wnt/β-catenin signaling pathway.

## Discussion

**Figure 6.**
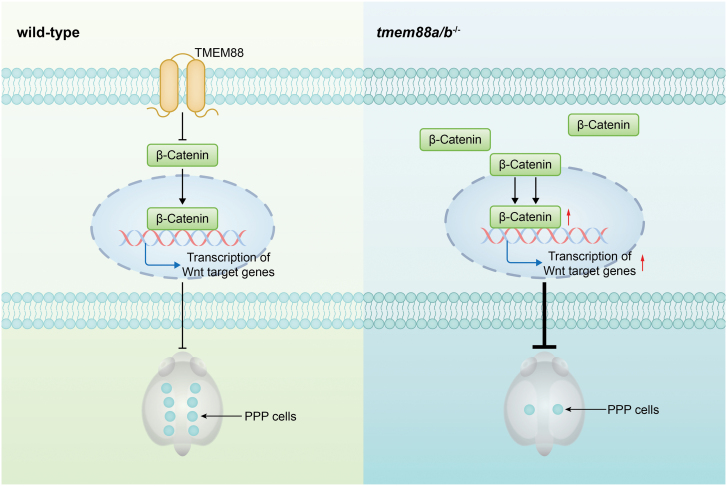
**A schematic diagram showing the regulatory mechanism, whereby TMEM88 promotes PPP specification through suppressing Wnt/β-catenin signaling.** The deficiency of *tmem88a/b* leads to an excessive accumulation of β-catenin in both the cytoplasm and nucleus of pharyngeal endodermal cells that are intended to differentiate into PPPs, thereby resulting in hyperactivation of Wnt signaling and defects in PPP specification.

In this study, we have made a significant discovery that the overactivation of the Wnt/β-catenin signaling pathway, either through pharmacological treatment or *tmem88a/b* depletion, disrupts the differentiation of pharyngeal endodermal cells into PPPs in zebrafish embryos. These findings underscore the critical role of Tmem88 in suppressing Wnt/β-catenin signaling for proper PPP specification (Fig. 6). Interestingly, previous studies have demonstrated the essential involvement of ectodermal BMP2b in activating Smad effectors in pharyngeal endodermal cells, which is necessary for PPP formation [9]. Notably, there is a growing body of evidence suggesting that the antagonistic activities of Wnt/β-catenin and BMP signals cooperate in regulating cell fate determination in various developmental processes, including embryonic dorsal–ventral patterning, spinal cord development, and the self-renewal and directional differentiation of intestinal stem cells [19–21]. Significantly, our previous and current studies have unequivocally shown that the suppression of intrinsic Wnt activity has no discernible impact on the process of PPP formation [9]. These insights prompt further investigation into whether BMP signaling in PPPs is up-regulated in response to overactivation of Wnt/β-catenin signaling in future studies.

While Wnt signaling has been implicated in the dynamic control of epithelial morphology during pouch morphogenesis [22], it is noteworthy that this morphogenetic function of Wnt signaling appears to be largely independent of β-catenin [22]. In contrast, our study establishes a robust connection between PPP specification and the Wnt/β-catenin axis. Consequently, the development of endodermal pouches involves a sequential orchestration of canonical (β-catenin-dependent) and non-canonical (β-catenin-independent) Wnt signaling pathways. Surprisingly, although *tmem88a/b* depletion led to a decrease in the emergence of PPPs during the early somite stages, pouch formation was not significantly affected at 36 hpf. Considering the involvement of Wnt/β-catenin signaling in cell proliferation and cell fate decisions in various stem/progenitor cells [23, 24], it is plausible that the loss of PPPs in *tmem88a/b*^−/−^ mutants is compensated by accelerated proliferation of residual precursor cells or the emergence of an alternative cell population during later developmental stages.

In this study, we have observed an aberrant activation of the Wnt/β-catenin signaling pathway in *tmem88a/b*-depleted PPPs. This finding raises a significant scientific question regarding the specific Wnt ligand responsible for initiating this cascade in the PPPs. Previous studies have indicated the expression of *wnt2bb* in the pharyngeal ectoderm between 20 hpf and 36 hpf, where it regulates the development of pharyngeal arch arteries [13]. Additionally, *wnt2bb* is expressed in the lateral plate mesoderm and plays a crucial role in the specification of liver precursors [25]. However, it remains unclear whether *wnt2bb* is expressed in the neighboring tissues of the pharyngeal endoderm during somitogenesis and whether it contributes to PPP specification. Further investigation is necessary to thoroughly explore these aspects.

## Research limitations

One limitation of our study is that we have not found the reason how the loss of PPPs in *tmem88a/b*^−/−^ mutants is compensated during later developmental stages. Another limitation in this study is that we have observed an aberrant activation of the Wnt/β-catenin signaling pathway in *tmem88a/b*-depleted PPPs. However, it is necessary to identify the Wnt ligand that is expressed specifically in the neighboring tissues of the pharyngeal endoderm during somitogenesis and to explore whether it contributes to PPP specification.

## Methods

### Research ethics

The zebrafish embryo studies were approved by the Animal Care and Use Committee at the Institute of Zoology, Chinese Academy of Sciences (permission number: IOZ-13048).

### Zebrafish strains

Wild-type embryos were obtained through natural mating of Tuebingen zebrafish. The embryos were raised in Holtfreter’s solution at a temperature of 28.5°C and staged based on their morphological characteristics. Homozygous mutants for *tmem88a*^*−/−*^, *tmem88b*^*−/−*^, and *tmem88a/b*^*−/−*^ were identified among the offspring of heterozygous mutant parents using genotyping techniques as previously described [13]. To visualize the development of pharyngeal pouches, we utilized transgenic embryos *Tg*(*sox17:GFP*), *Tg*(*nkx2.3-mCherry*), and *Tg*(*sox17:GFP;nkx2.3-mCherry*). Furthermore, *Tg*(*hsp70l:dkk1b-EGFP*) transgenic embryos were employed to inhibit the Wnt signaling pathway.

### RNA synthesis, morpholinos, and microinjection

Capped mRNAs for *tmem88a* and *tmem88b*, including their variants with or without the VWV motif, were synthesized *in vitro* from the respective linearized plasmids using the mMessage mMachine kit (Ambion). For the generation of antisense RNA probes, the MEGAscript Kit (Ambion) was utilized, and the probes were labeled with Digoxigenin-UTP. The *in vitro* transcription process adhered to the instructions provided by the manufacturer.

### Whole-mount *in situ* hybridization

Whole-mount *in situ* hybridization was performed following standard procedures with the NBT-BCIP substrate. For two-color fluorescence *in situ* hybridization, anti-digoxigenin-POD (11633716001, Roche) and anti-fluorescein-POD (11426346910, Roche) antibodies were utilized as primary antibodies to detect the digoxigenin-labeled mCherry probe and the fluorescein-labeled *tmem88a* and *tmem88b* probes, respectively. Subsequently, fluorescence *in situ* hybridization was carried out using the Perkin Elmer TAS fluorescein system (NEL701A001KT) following the manufacturer’s instructions.

### Immunostaining and confocal microscope

The embryos were fixed overnight in 4% paraformaldehyde. Subsequently, they underwent four rinses with phosphate-buffered saline with Tween 20 (PBST) at 5-min intervals. Following the rinses, the embryos were blocked with 10% heat-inactivated goat serum at room temperature for 1 h. Affinity-purified primary antibodies, including anti-β-catenin antibody (1:500; ab6302, Abcam), anti-Zn8 antibody (1:500; ZDB-ATB-081002-19, Zebrafish International Resource Center), and anti-mCherry antibody (1:1000; ab125096, Abcam), were then added and allowed to incubate overnight at 4°C. Afterward, the samples were washed three times with PBST and incubated with secondary antibodies: DyLight 488-conjugated Goat anti-rabbit IgG (1:200; 711-545-152, Jackson) and DyLight 594-conjugated Goat anti-mouse IgG (1:200; 715-585-150, Jackson) for 1 h at room temperature. In specific experiments, DAPI (1:10,000, Sigma) was used for nuclear staining. Finally, the stained embryos were embedded in 2% low melting agarose and imaged using a Nikon A1R^+^ confocal microscope with consistent settings.

### Pharmacological treatment

Pharmacological treatment was conducted by incubating embryos in the dark in Holtfreter’s solution containing CCT036477 (SML0151, Sigma–Aldrich) or BIO (B1686, Sigma–Aldrich) to inhibit or activate Wnt signal activity. The embryos were treated with CCT036477 or BIO from the bud stage until the 17 ss.

### Confocal imaging

The embryos were anesthetized and then embedded in 1% low-melt agarose (0815, AMRESCO) at specified time points for live imaging using a Nikon A1R^+^ confocal microscope. All acquired confocal stack images were subsequently processed using Nikon NIS-Elements AR 4.13.00 software.

### Statistical analysis

The results were analyzed using GraphPad Prism 9 software and are presented as the mean ± standard deviation (SD). Differences between the control and treated groups were analyzed using the unpaired two-tailed Student’s *t*-test. Statistical significance was defined as *P* < 0.05.

## Data availability

The data supporting the findings of this study are available within the article and its supplementary materials.
